# Role of Exosomal Non-coding RNAs in Gastric Cancer: Biological Functions and Potential Clinical Applications

**DOI:** 10.3389/fonc.2021.700168

**Published:** 2021-06-14

**Authors:** Feng Hu, Jixuan Liu, Huibo Liu, Fan Li, Minjie Wan, Manli Zhang, Yanfang Jiang, Min Rao

**Affiliations:** ^1^ Department of Gastroenterology, The First Hospital of Jilin University, Changchun, China; ^2^ Department of Pathology, The First Hospital of Jilin University, Changchun, China; ^3^ Department of Dermatology, The First Hospital of Jilin University, Changchun, China; ^4^ Department of Anesthesia, The First Hospital of Jilin University, Changchun, China; ^5^ Department of Central Laboratory, The First Hospital of Jilin University, Changchun, China; ^6^ Key Laboratory of Organ Regeneration & Transplantation of the Ministry of Education, Genetic Diagnosis Center, The First Hospital of Jilin University, Changchun, China

**Keywords:** non-coding RNAs, exosomes, gastric cancer, molecular mechanism, biomarker, cancer therapy

## Abstract

Gastric cancer (GC) is one of the most common fatal cancers worldwide. The communication between GC and other cells in the GC microenvironment directly affects GC progression. Recently, exosomes have been revealed as new players in intercellular communication. They play an important role in human health and diseases, including cancer, owing to their ability to carry various bioactive molecules, including non-coding RNAs (ncRNAs). NcRNAs, including micro RNAs, long non-coding RNAs, and circular RNAs, play a significant role in various pathophysiological processes, especially cancer. Increasing evidence has shown that exosomal ncRNAs are involved in the regulation of tumor proliferation, invasion, metastasis, angiogenesis, immune regulation, and treatment resistance in GC. In addition, exosomal ncRNAs have promising potential as diagnostic and prognostic markers for GC. Considering the biocompatibility of exosomes, they can also be used as biological carriers for targeted therapy. This review summarizes the current research progress on exosomal ncRNAs in gastric cancer, focusing on their biological role in GC and their potential as new biomarkers for GC and therapeutics. Our review provides insight into the mechanisms involved in GC progression, which may provide a new point cut for the discovery of new diagnostic markers and therapeutic strategies.

## Introduction

Gastric cancer (GC), one of the most common fatal cancers, is the fifth most commonly diagnosed tumor and the fourth most common tumor-related cause of death worldwide. More than one million new GC cases (approximately 5.6% of confirmed cancer cases) and nearly 770,000 GC-related deaths (approximately 7.7% of cancer deaths) have been reported in 2020 (1). Although tremendous advances have been made in the diagnosis and treatment of GC, its prognosis remains unsatisfactory with the statistics of more than 70% of patients with GC eventually die from this disease (2). Furthermore, resistance to treatment is a major clinical problem, which partially leads to the unsatisfactory survival rate of GC patients. In addition to the rapid proliferation of GC cells, extensive metastasis, genetic heterogeneity of tumors, and treatment resistance, the poor prognosis of GC patients stems from the limited understanding of the molecular pathogenesis of GC and the lack of timely diagnostic and sensitive monitoring tools. Therefore, it is essential to extend knowledge regarding the molecular mechanism of GC progression, explore reliable GC biomarkers, and develop therapeutic targets.

During the occurrence and development of GC, cancer cells not only proliferate and invade, but also interact with other cells, including stromal cells, to establish a microenvironment conducive to their growth and metastasis (3). Increasing evidence indicates that the communication between tumor cells and other cells in the GC microenvironment may directly affect various hallmark features of GC, such as tumor proliferation, invasion, metastasis, tumor angiogenesis, and tumor immune regulation (4). There are several ways in which tumor cells communicate to adjacent or distant cells, either by direct interaction between cell membrane receptors and ligands, or by the release of soluble molecules, including growth factors, cytokines, and chemokines, or by exosomes, which represents the additional way of cell communication that have been identified recently (5–7). Recently, as new players in cell-to-cell communication, exosomes have attracted much attention because that exosomes can target recipient cells through their surface molecules, and then deliver various biologically active molecules (such as proteins, nucleic acids and lipids) to the recipient cells through a variety of mechanisms, thereby modifying the physiological state of the recipient cell (8, 9). Among these biologically active components, non-coding RNAs (ncRNAs), such as microRNAs (miRNAs), long non-coding RNAs (lncRNAs), and circular RNAs (circRNAs), are enriched and stabilized in exosomes. These components have aroused widespread attention for their regulatory functions in various cancers, such as breast cancer, colon cancer, lung cancer, pancreatic cancer, and GC (10). In this review, we summarize the latest studies on the role of exosomal ncRNAs in GC progression and their potential clinical applications. Our review provides insight into the mechanisms involved in GC progression, which may provide a new point cut for discovering new diagnostic markers and therapeutic strategies.

This article reviews the role of exosomal ncRNAs in the progression of GC and their potential clinical applications. In terms of GC progression, we mainly discuss the role of exosomal ncRNAs in GC proliferation, invasion, metastasis, angiogenesis, immune regulation, and therapeutic resistance. In terms of the potential clinical applications of GC, we mainly explained the potential value of exosomal ncRNAs in GC in terms of promising biomarkers (mainly diagnostic and prognostic markers) and treatment strategies. This review will help researchers in clinical oncology and provide a conceptual framework for future studies investigating the role of exosomal ncRNAs as potential biomarkers and novel or alternative treatment options, overcoming the limitations of conventional GC therapy.

## Biogenesis and Characteristics of ncRNAs

According to their size, ncRNAs can be divided into small RNAs (mainly miRNAs) and long RNAs (including lncRNAs and circRNAs) (11). In general, miRNAs and lncRNAs are generated from primary transcripts *via* linear splicing. MiRNAs are short single-stranded RNAs, approximately 20 to 22 nucleotides in length. They primarily regulate mRNA expression primarily by binding to the 3′-untranslated regions of target genes to promote mRNA degradation or inhibit translation, although this mRNA silencing can be reversed (12). A single miRNA can potentially target and can be regulated by multiple mRNAs. Therefore, miRNAs exhibit a complex regulatory mechanism of post-transcriptional gene expression, which may have synergistic effects on many cellular processes (13).

LncRNAs are a group of non-coding RNAs that longer than 200 nucleotides in length (14) that participate in the regulation of gene expression at the epigenetic, transcriptional, and post-transcriptional levels *via* their interaction with DNA, RNA, or proteins (15).

CircRNAs, which differ from miRNAs and lncRNAs, are a new type of ncRNAs with a characteristic covalent closed-loop structure that recently gained a new research hotspot (16, 17). Increasing evidence has shown that circRNAs regulate gene expression at the transcriptional, post-transcriptional, and translational levels. They also participate in many pathological processes by regulating alternative splicing, sponging miRNAs, chelating functional proteins, and even encoding proteins (18). The distinct closed-loop structure of circRNA makes it highly resistant to exonucleases. Compared with linear RNAs, circRNAs possess more stable characteristics and longer half-lives (17, 19). Therefore, circRNAs are more suitable than linear RNAs as biomarkers (20).In addition to regulating the gene expression of the cells where ncRNAs are located (18, 21–27), ncRNAs can perform regulatory functions when they are selectively packaged into exosomes and transported to other cells (10).

## Biogenesis and Characteristics of Exosomes

**Figure 1:**
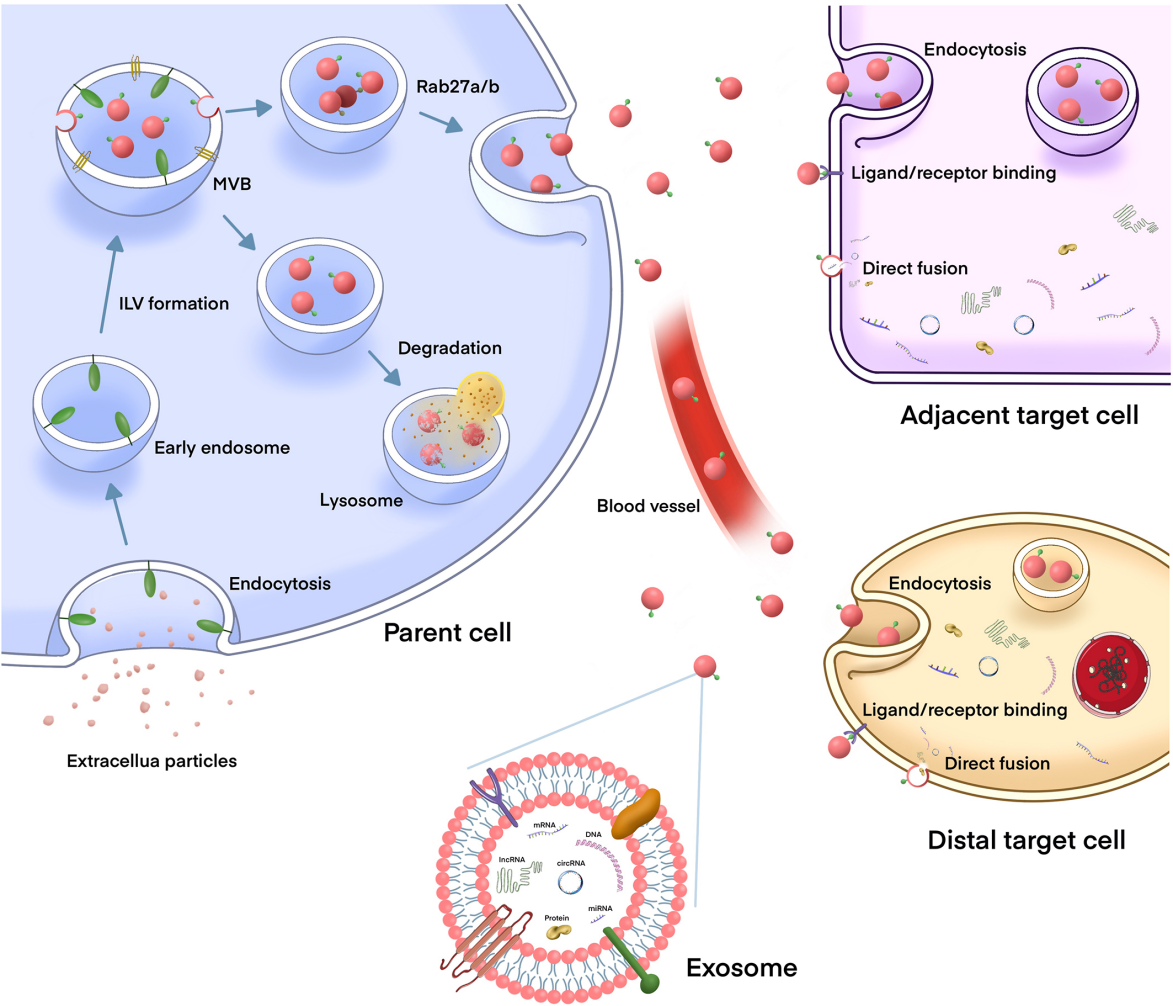
The biogenesis and release of exosomes, and their role in intercellular communication. The biogenesis of exosome is initiated by the membrane invagination of early endosomes, and then form mature intracellular multivesicular bodies (MVBs), which organize intraluminal vesicles (ILVs) *via* endosomal sorting complex required for transport (ESCRT) dependent or independent pathways. MVBs can either fuse with lysosomes to degrade, or fuse with plasma membrane to release exosomes into the extracellular environment. After being released, the exosomes can play a biological role either locally or distant sites which is through the circulation. Exosomes are taken up by target cells in different mechanisms, such as endocytosis, direct fusion with the plasma membrane or ligand/receptor interaction.Then, exosomes release their contents (including DNAs, proteins, mRNAs, miRNAs, lncRNAs, and circRNAs) to the recipient cells to regulate cell-to-cell communication.

Exosomes are phospholipid bilayer nanovesicles with a diameter of 30–150 nm and contain a series of molecules, including proteins, lipids, and different types of nucleic acids (10, 28). They are secreted by a variety of cells, including cancer cells, and exist t in almost all body fluids (29, 30). The biogenesis of exosomes is initiated by the membrane invagination of early endosomes, and then form mature intracellular multivesicular bodies (MVBs), which organize intraluminal vesicles (ILVs) *via* endosomal sorting complex required for transport (ESCRT) dependent or independent pathways (31). Several mechanisms of exosome formation independent of ESCRT have been identified; however, ESCRT is one of the most important mechanisms, playing an important role in ILV and MVB formation (32, 33). Generally, the MVBs either are degraded by lysosomes or fuses with the plasma membrane to secrete ILVs (exosomes) into the extracellular environment. The exosomes release is regulated under the Rab GTPases 27A/B molecules, which are related to the transportation of MVBs to specific regions of the cell membrane (32).Then, exosomes are taken up by adjacent or distant recipient cells in various ways, including receptor/ligand binding, direct membrane fusion, and internalization *via* endocytosis, to play a regulatory role in these cells (34) (**Figure 1**).

Exosomes are secreted by almost all types of cells in the human body (35). They regulate a variety of pathophysiological processes, including intercellular communication (36–39), immune regulation (40, 41), inflammation (42), tumor growth (43), and infection (44, 45). Increasing evidence shows that exosomes are involved in the pathogenesis of various diseases, such as cancer (46), cardiovascular diseases (47), and neurological diseases (48). Due to the biological functions of exosomes and their extensive contributions to various diseases, they have attracted widespread attention. First, exosomes carrying surface molecules can interact with target cells to activate cell signals and participate in cell-to-cell communication (9, 49). Secondly, exosomes can transfer the biologically active substances they carry to the recipient cells and change the physiological state of the recipient cells (50, 51). Finally, exosomes have potential clinical applications as disease biomarkers and therapeutic delivery vehicles for currently incurable diseases (37, 52).

## Biological Functions of Exosomal ncRNAs in GC

**Figure 2:**
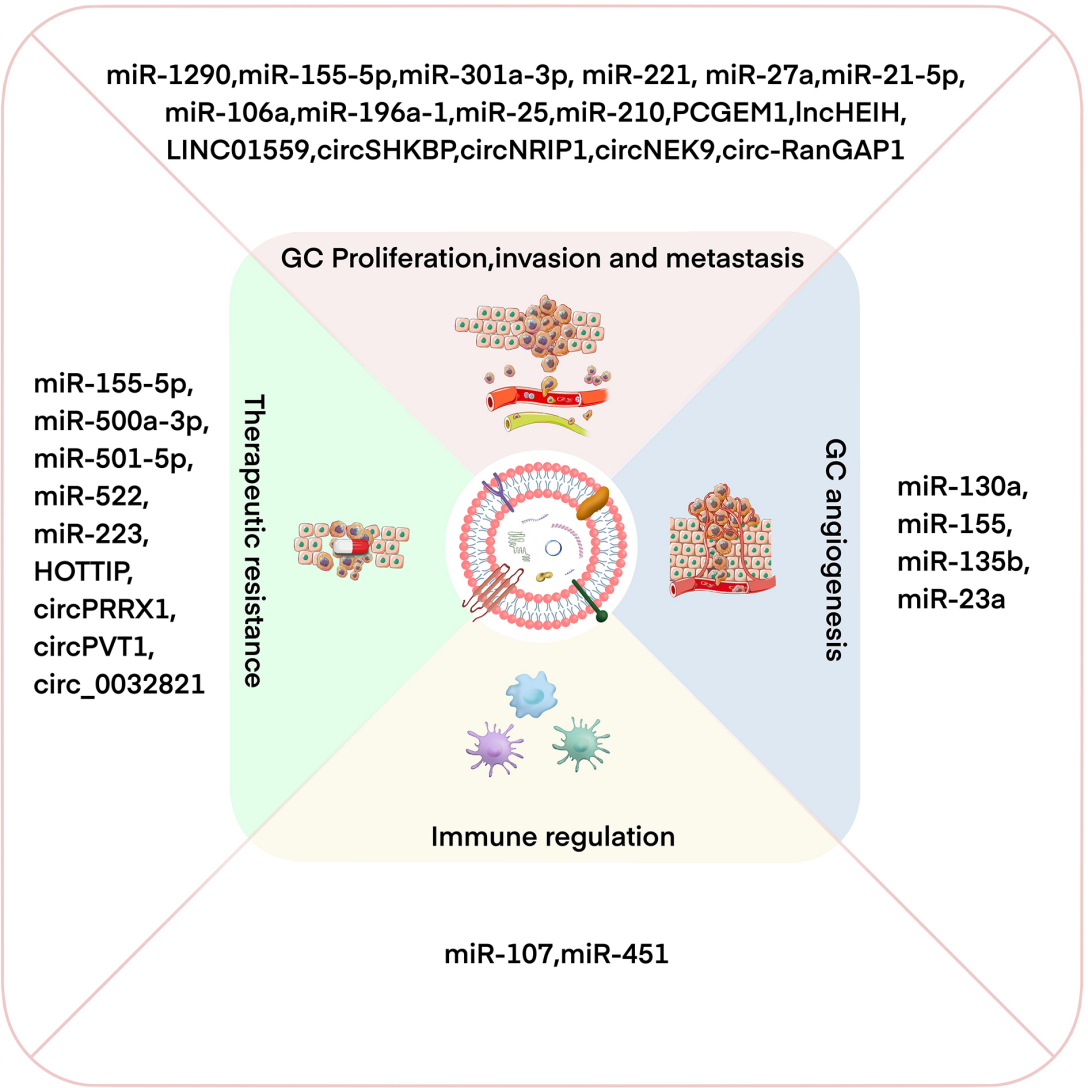
Exosomal ncRNAs have important effects on regulating GC proliferation/invasion/metastasis, angiogenesis, immune regulation, and therapeutic resistance.

GC is a common and fatal tumor, and its underlying molecular mechanism remains unclear because of the complexity of the disease mechanism and the limitations of the current scientific research level. Increasing evidence shows that exosomes participate in the occurrence and development of GC by transporting biologically active molecules (such as ncRNAs) between cells (46, 53). Exosomal miRNA is expressed differently in different cell and tissue types and plays a crucial role in the occurrence and development of GC (54, 55). Moreover, the availability, abundance, and stability of exosomal miRNAs in biological fluids make them potentially ideal biomarkers for various types of cancer, including GC (56). In addition, lncRNAs can be selectively distributed among exosomes and contribute to cell-to-cell communication within the tumor microenvironment (TME) (57). Exosomal lncRNAs are also involved in the progression of GC and can be potential biomarkers and therapeutic targets in GC (58). Recent studies have found that circRNAs are also of abundance in exosomes and that they are transported to recipient cells by exosomes to exert various biological effects (59, 60). Exosomal ncRNAs play a key role in many aspects of GC biology, including proliferation, invasion, metastasis, angiogenesis, immune regulation, and therapeutic resistance (10) (**Figure 2**). In the following sections, we summarize the current research progress on the specific roles and mechanisms of exosomal ncRNAs in GC progression (**Table 1**).

**Table 1:** The biological function of exosomal ncRNAs in GC.

NcRNAs	Parent cell	Target cell	Biological function	reference
miR-1290	GC cells	GC cells	Promote proliferation, migration and invasiveness	(61)
miR-155-5p	GC cells	GC cells	Promote proliferation and migration	(62)
miR-301a-3p	GC cells	GC cells	Promote proliferation, invasion, migration and EMT	(63)
miR-221	BM-MSCs	GC cells	Promote proliferation, migration, invasion, and adhesion to the matrix	(64)
miR-221	GC-MSCs	GC cells	proliferation and migration	(65)
miR-25, miR-210	esophageal adenocarcinoma cells	Gastrointestinal cells	Promote proliferation and cellular viability, inhibit apoptosis	(66)
miR-27a	GC cells	GC cells and HSF-1 cells	Promote proliferation, motility and metastasis, induce fibroblasts transformation into CAFs	(67)
miR-21-5p	GC cells	PMCs	Promote MMT of PMCs and peritoneal metastasis	(68)
miR-106a	GC cells	PMCs	Promote peritoneal metastasis	(69)
miR-196a-1	high-invasive GC cells	Low-invasive GC cells	Promote invasion and metastasis	(70)
lncRNA PCGEM1	Hypoxic GC cells	Normoxic GC cells	Promote invasion and migration, EMT	(71)
lncHEIH	GC cells	Normal gastric cells	Promote proliferation and migration	(72)
LINC01559	MSCs	GC cells	Promote proliferation, migration and stemness	(73)
circSHKBP	GC cells	GC cells	Promote proliferation, migration, invasion and angiogenesis	(74)
circNRIP1	GC cells	GC cells	proliferation, migration and invasiveness	(59)
circNEK9	GC cells	GC cells	migration and invasion	(75)
circ-RanGAP1	GC cells	GC cells	migration and invasion	(76)
miR-130a	GC cells	Endothelial cells	promote angiogenesis and tumor growth	(54)
miR-155	GC cells	Endothelial cells	promote angiogenesis and tumor growth	(77)
miR-155	GC cells	Endothelial cells	promote angiogenesis	(78)
miR-135b	GC cells	Endothelial cells	promote angiogenesis	(79)
miR-23a	GC cells	Endothelial cells	promote angiogenesis	(80)
miR-107	GC cells	MDSCs	Promote immunosuppression microenvironment,	(81)
miR-155-5p	PTX-resistant GC cells	PTX-sensitive GC cells	Promote EMT and chemoresistance to PTX	(82)
miR-500a-3p	DDP-resistant GC cells	parental GC cells	Promote chemoresistance to DDP and stemness	(83)
miR-501-5p	ADR- resistant GC cells	parental GC cells	Promote chemoresistance to ADR, proliferation, migration and invasion	(84)
miR -522	CAFs	GC cells	Promote chemoresistance to PTX and DDP	(85)
miR-223	macrophages	GC cells	Promote chemoresistance to ADR	(86)
HOTTIP	DDP-resistant GC cells	Parental GC cells	Promote chemoresistance to DDP	(87)
circPRRX1	ADR- resistant GC cells	Parental GC cells	Promote chemoresistance to ADR	(88)
circPVT1	DDP-resistant GC cells	Parental GC cells	Promote chemoresistance to DDP	(89)
circ_0032821	OXA-resistant GC cells	OXA-sensitive GC cells	Promote chemoresistance to DDP, proliferation, migration, and invasion	(90)

### Exosomal ncRNAs and GC Proliferation, Invasion, and Metastasis

Some miRNAs can be selectively packaged into exosomes and regulate GC proliferation, invasion, and metastasis. For instance, miR-1290 is overexpressed in many malignant tumors, including GC, and promotes tumor cell proliferation, invasion, and chemotherapy resistance (91–93). Huang et al. also reported that miR-1290 is overexpressed in both GC patients and cell lines. MiR-1290 could be transported by exosomes derived from GC cells to the surrounding cells to promote tumor proliferation, migration, and invasion by directly targeting NKD1, an important negative regulator in GC (61). Furthermore, Shi et al. observed that the miR-155-5p levels in the exosomes from tissues of GC patients and GC cell lines were substantially increased. MiR-155-5p was delivered to GC cells by exosomes to promote their proliferation and migration by targeting *TP53INP1* (a tumor suppressor gene) (62). In addition, recent studies have found that GC cells can produce exosomes that are rich in miR-301a-3p in the hypoxic TME. For example, exosomal miR-301a-3p can be transmitted between GC cells to inhibit HIF-1α degradation by targeting PHD3, thereby promoting the proliferation, invasion, migration, and epithelial-mesenchymal transition (EMT) of GC (63). At present, the role of miR-221 in promoting tumor cell proliferation, migration, and drug resistance has been widely recognized (94, 95). Two independent studies have revealed that exosomes can deliver miR-221 to GC cells and promote their proliferation and migration; however, these exosomes have different origins, i.e., bone marrow mesenchymal stem cells (BM-MSCs) and GC tissue-derived mesenchymal stem cells (GC-MSCs) (64, 65). Interestingly, Ke et al. reported that exosomal miR-25 and miR-210 derived from other tumors, such as esophageal adenocarcinoma, can also promote proliferation and cellular viability in gastroids (66)

Furthermore, exosomal miRNAs can act on GC cells and other cells in the TME, such as CAFs and peritoneal mesothelial cells (PMCs). The malignant biological behavior of GC is not only determined by the tumor cells themselves but is also regulated by the TME. Fibroblasts are one of the main components of stromal cells and have been identified as an essential component in tumor progression (96). Previous studies have reported that the miR-27a located on chromosome 19 plays a vital role in tumor occurrence and development (97). Wang et al. found that the level of miR-27a in GC cells-derived exosomes is significantly increased. MiR-27a functions as an oncogene that not only promotes the proliferation, motility, and metastasis of cancer cells but also induces the transformation of fibroblasts into cancer-associated fibroblasts (CAFs) both *in vitro* and *in vivo* (67). In approximately 50% of GC cases, local or distant metastasis occurred at the time of initial diagnosis (98). Despite surgery and chemotherapy, peritoneal metastasis occurs in more than half of patients with GC (99, 100). PMCS in the peritoneal cavity can undergo mesothelial-to-mesenchymal transition (MMT), a crucial t morphological change in peritoneal metastasis (101) to promote early cancer metastasis (102–104). Recent studies have reported that exosome l miRNAs promote cancer metastasis (105). For example, miR-21-5p and miR-106a can be delivered to PMCs by GC-derived exosomes, thus inducing the MMT of PMCs and promoting tumor peritoneal metastasis. Interestingly, they all promote peritoneal metastasis by targeting Smad7 (68, 69).

In addition to the peritoneal cavity, the liver is a common site for distant metastasis in advanced GC, which seriously impairs the quality of life and survival of GC patients. Feng et al. found that exosomes containing miR-196a-1 are secreted by highly invasive GC cells and can be taken up by low-invasive GC cells. Ectopic miR-196a-1 expression promotes the invasiveness of low-invasive GC cells by targeting SFRP1 (a secretion antagonist of the Wnt/β-catenin signaling pathway) to stimulate liver metastasis both *in vivo* and *in vitro* (70). Overall, the miRNAs above mentioned promote the proliferation, invasion, and metastasis of GC.

In addition to miRNAs, some exosomal lncRNAs that regulate the proliferation, invasion, and metastasis of GC have been identified, such as lncRNA PCGEM1 (71), lncHEIH (72), and LINC01559 (73). Hypoxia is an important feature of the TME and facilitates the progression of GC (63). Piao et al. cultured GC cells at 1% O_2_ (hypoxia-cultured GC cells, HGC) and 20% O_2_ (normoxic-cultured GC cells, NGC) and found that the HGC medium promotes the malignant phenotype of NGC. The lncRNA prostate cancer gene expression marker 1 (PCGEM1) was transmitted to NGC by HGC-derived exosomes and maintained the stability of SNAI1 and suppressed its degradation, which could induce the EMT of GC and promote its invasion and migration (71). In addition, Liu et al. reported that lncHEIH is upregulated in GC tissues and cell lines and is positively correlated with high-expression levels of EZH2. Moreover, exosomal lncHEIH was released by GC cells and then absorbed by normal gastric cells. The absorption of lncHEIH contributed to the up-regulation of EZH2, which inhibited the expression of GSDME (a tumor-suppressor gene) *via* the methylation of its promoter, thereby promoting the malignant transformation of normal gastric cells  (72).

Mesenchymal stem cells (MSCs) are efficient and prolific producers of exosomes (106), MSC-derived exosomes have recently gained increasing research attention because they play an important role in tumorigenesis, angiogenesis, and metastasis (107). For example, Wang et al. detected the upregulated expression of LINC01559 in GC tissues, which was lower in GC cells than in MSCs. They revealed that LINC01559 is transported from MSCs to GC cells *via* exosomes. Mechanistic studies showed that LINC01559 activates the PI3K/AKT pathway by sponging miR-1343-3p to upregulate phosphoglycinate kinase 1(PGK1). On the other hand, LINC01559 recruits EZH2 to the PTEN promoter to induce the methylation of the latter, thereby resulting in PTEN inhibition. Notably, LINC01559 targets both PGK1 and PTEN and promotes GC progression by activating the PI3K/AKT pathway (73).

In addition to miRNAs and lncRNAs, exosomal circRNAs that play essential roles in the proliferation, invasion, and metastasis of GC have been reported, such as circSHKBP1 (74), circNRIP1 (59), circNEK9 (75), and circ-RanGAP1 (76). Xie et al. observed the increased expression of circSHKBP1 in both GC tissues and serum, but the decreased level of exosome circSHKBP1 following gastrectomy. More importantly, upon the ectopic expression of circSHKBP1 in GC cells, circSHKBP1 was increasingly loaded into exosomes, thereby promoting the proliferation, migration, and invasion of GC cells.Mechanistically, circSHKBP1 increased the expression of HUR and enhanced the stability of VEGF mRNA by sponging miR-582-3p. In addition, circSHKBP1 directly binds to HSP90 to inhibit the interaction between STUB1 and Hsp90 and the ubiquitination of HSP90, thus leading to the development of GC both *in vivo* and *in vitro* (74). Another study found that circNRIP1 is highly expressed in GC cells. *In vitro*, circNRIP1 can be transmitted between GC cells *via* exosome communication to further regulate AKT1 expression and EMT by targeting miR-149-5p, thereby promoting the proliferation, migration, and invasion of GC cells and tumor metastasis *in vivo* (59). In addition, circNEK9 and circ-RanGAP1 were significantly upregulated in both tissues and plasma exosomes of GC patients. The plasma exosomal circNEK9 and circ-RanGAP1 promoted the migration and invasion of recipient GC cells to subsequently promote GC progression through the miR-409-3p/MAP7 and miR-877-3p/VEGFA axes, respectively (75, 76). In summary, these studies demonstrate that the selective secretion and transmission of ncRNAs between cells through exosomes play significant roles in regulating the proliferation, invasion, and metastasis of GC.

### Exosomal ncRNA and GC Angiogenesis

The poor prognosis of GC is partially attributable to its continued proliferation and metastasis due to enhanced angiogenesis. Tumor angiogenesis is a complex, multi-step process that depends on the synergistic effects of multiple regulators (108, 109). Angiogenesis is involved in the entire cancer pathophysiology, including occurrence (110), progression (111), invasion, and metastasis (112, 113). However, at present, the underlying mechanisms of angiogenesis have yet to be fully understood. Therefore, it is of necessity to elucidate the molecular mechanisms underlying tumor angiogenesis to develop more effective anti-angiogenesis therapies.

Many researchs have indicated that ncRNAs play an important regulatory role in tumor angiogenesis (114, 115). In the TME, endothelial cells can capture tumor-derived exosomes, promoting the growth of new blood vessels (116). Increasing evidence shows that the delivery of ncRNAs in the GC microenvironment by exosomes partially regulates angiogenesis (117). As a tumor miRNA, mi-130a is involved in different pathological mechanisms in various tumors (118). Yang et al. found that the expression of miR-130a is significantly increased in GC cells and their exosomes. MiR-130a in GC cells is delivered into vascular cells by exosomes and directly targets c-MYB to promote angiogenesis and tumor growth *in vivo* and *in vitro*  *(* 54). In addition, GC-cell-derived exosomes can be used as carriers to deliver miR-155 to vascular endothelial cell. The miR-155 of GC-derived exosomes can targets c-MYB, increase the expression of VEGF, promote the growth, metastasis, and tubular formation of vascular cells, thus causing the occurrence and development of tumors (77). The c-MYB plays an irreplaceable physiological role in vascular maturation and the maintenance of vascular homeostasis, and its imbalance is associated with cancer development (119, 120). Coincidentally, miR-130a and miR-155 both promote tumor angiogenesis by targeting c-MYB.

Zhou et al. also reported that GC-derived exosomal miR-155 is the driver of angiogenesis *via* different experimental methods (78). MiR-155 can be released from GC cells by exosomes and proceeds to be absorbed by vascular endothelial cells. miR-155 can target the forkhead box O3 (FOXO3a) to inhibit its protein expression and promote the formation of new GC blood vessels *in vivo* and *in vitro*, thus promoting the progression of GC. Bai et al. revealed the negative correlation between miR-135b expression and FoxO1, i.e., miR-135b is highly expressed in GC tissues and plasma exosomes, whereas FoxO1 is weakly expressed. Moreover, miR-135b can be secreted by exosomes from GC cells and then delivered to endothelial cells to inhibit the expression of FOXO1 protein, promote blood vessel growth, and exert carcinogenic effects (79). FOXO proteins, which belong to the forkhead box O superfamily, play a significant role in tumorigenesis and development (121). To date, four subfamily members have been identified in mammals: FoxO1, FoxO3, FoxO4, and FoxO6 (122). Interestingly, both miR-135b and miR-155 can promote tumor blood vessel growth by inhibiting the FOXO protein expression.

MiR-23a is one of the most widely studied miRNAs in different types of human cancers and plays a major role in tumor initiation and development (123). Du et al. found that exosomes released by GC promote angiogenesis by delivering miR-23a to endothelial cells. Furthermore, miR-23a can target PTEN, upregulate VEGF expression, and down-regulate the expression of TSP-1, thus promoting GC angiogenesis by providing blood supply for cell growth (80). These studies have demonstrated that GC cells can promote endothelial angiogenesis through exosomal ncRNAs.

### Exosomal ncRNA and GC Immune Regulation

As an important component of the GC microenvironment, GC-related immune cells directly or indirectly affect GC progression by regulating the immune response (4). GC cells can interact with immune cells through exosomes to create an optimal TME for tumor growth (50, 51). Therefore, targeting these immune cells and their associated molecules represents a novel and promising treatment strategy for patients with GC.

Myeloid-derived suppressor cells (MDSCs) are a heterogeneous population of immature myeloid cells (124) that promote tumor escape in the TME by inhibiting T cell response and catalyzing the production of other immunosuppressive cells such as Treg to support tumor growth and survival  (125, 126). Previous studies have revealed that exosomes and miRNAs are involved in the expansion, activation, and functional regulation of MDSCs in the TME (127–129). Particularly, Ren et al. found that miR-107 is highly expressed in GC cells and GC-derived exosomes. MiR-107 can be taken up by MDSCs through GC-derived exosomes. Furthermore, miR-107 can target and inhibit the expression of *Dicer1* and *PTEN*, induce the accumulation and proliferation of MDSCs, and promote the progression of GC. Therefore, miR-107 in GC-derived exosomes may participate in GC progression by regulating MDSCs and may become a new target for GC treatment (81). In addition, miR-451 derived from GC exosomes under low-glucose conditions decreases the level of the 5′amp activated protein kinase of T cells, thereby increasing mTOR activity and promoting Th17 differentiation. These findings strengthen our understanding of how tumor cells change the TME through exosomal ncRNAs (130). These studies show that GC cells can affect the accumulation and proliferation of MDSCs and the differentiation of Th17 through exosomal miRNAs, thereby affecting the tumor immune environment.

### Exosomal ncRNA and GC Chemotherapy Resistance

Currently, chemotherapy after surgery is one of the main treatment methods used to treat GC. However, chemoresistance remains a major obstacle in the successful treatment of cancer. Recently, increasing evidence has shown that as an important communicator between cells, exosomes participate in the chemoresistance of tumors, ultimately leading to cancer treatment failure (131–133). In addition, recent studies have found that the delivery of ncRNA by exosomes is essential in promoting the adaptability of tumor cells to the microenvironment and chemoresistance (134, 135). Therefore, exploring the mechanism of chemoresistance is of great significance to improving the poor prognosis of cancer and successfully treating cancer.

Paclitaxel (PTX), cisplatin (DDP), and doxorubicin (ADR) are the first-line drugs extensively used in anti-tumor chemotherapy. Although they exert powerful and broad anti-tumor activities, their efficacy is limited by drug resistance that is inevitably acquired after long-term exposure (136–139). Wang et al. concluded that miR-155-5p is more highly expressed in the PTX-resistant GC cell line (MGC-803R) than in the PTX-sensitive GC cell line (MGC-803S). The exosomes derived from MGC-803R cells transferred miR-155-5p to MGC-803S, which underwent EMT and showed drug-resistant phenotypes. Furthermore, miR-155 5p induces malignant and chemoresistant phenotypes by targeting GATA3 and TP53INP1 (tumor suppressors) (82). Thus, targeting miR-155-5p may be a promising strategy to overcome PTX resistance in GC.

In addition, Lin et al. found that the expression levels of miR-500a-3p were increased in DDP-resistant GC cells (MGC803/DDP) and their secreted exosomes compared with their corresponding parental cells. MiR-500a-3p was delivered to the recipient cell MGC803 through exosomes derived from MGC803/DDP to enhance its DDP resistance and stemness *in vitro* and *in vivo* by targeting FBXW7 (83). Liu et al. reported that the miR-501-5p level in adriamycin-resistant GC SGC7901/ADR Exo (ADR Exo) is higher than in SGC7901 cells (7901 Exo). ADR Exo can be absorbed by SGC7901 and transfer miR-501-5p to SGC7910 cells. The exosomal miR-501-5p targeting of BLID (a new tumor-suppressor) promotes the proliferation, migration, invasion, and ADR resistance and suppresses apoptosis in recipient cells *via* Akt phosphorylation and caspase-9/-3 inactivation. *In vivo* tests further verified that exosomal miR-501-5p induced ADR resistance and promoted GC tumorigenesis (84).

Exosomes produced by other cells in the TME, such as CAF and M2 macrophages, can also induce chemoresistance in GC cells (85, 86, 140). Ferroptosis is a novel mode of non-apoptotic cell death induced by the accumulation of toxic lipid peroxides (lipid-ROS) in an iron-dependent manner (141). Ferroptosis plays a vital role in mediating tumor progression and drug resistance in multiple tumors, such as liver cancer, lung cancer, and colon cancer (142–144). Recently, the release of exosome-miR-522 mainly derived from CAFs in the TME has been revealed to be activated by cisplatin and paclitaxel. Its delivery to GC cells resulted in ALOX15 suppression and decreased lipid-ROS accumulation, thus promoting chemoresistance (85). Zheng et al. found that M2-polarized macrophage-derived exosome microRNA-21 can transfer to GC cells to inhibit cell apoptosis by downregulating PTEN and enhancing the activation of the PI3K/AKT signaling pathway, thereby inducing DDP resistance in GC patients (140). In addition, Gao and colleagues reported that macrophage-derived exosomes transferred miR-223 into co-cultured GC cells to activate their resistance to ADR by targeting FBXW7, a p53-dependent tumor-suppressor protein (86).

In addition to miRNAs, exosomal lncRNAs are involved in inducing resistance to GC chemotherapy. Wang et al. observed that HOTTIP levels are upregulated in DDP-resistant GC cells and that its downregulation enhances DDP sensitivity. HOTTIP can be transmitted to sensitive cells by exosomes derived from DDP-resistant GC cells, thereby activating DDP resistance. Furthermore, HOTTIP functions as a ceRNA and activates HMGA1 in GC cells by sponging miR-218, thus promoting cisplatin resistance. Moreover, its high-expression in the serum has been associated with adverse reactions to DDP therapy in GC patients (87).

Exosomal circRNAs also contribute to GC resistance to chemotherapy. Wang et al. reported that the expression levels of circPRRX1 are increased in ADR-resistant GC cell lines. Exosomal circPRRX1 secreted by these resistant GC cells can be internalized by sensitive gastric cancer cells to enhance their resistance to ADR. The mechanism by which circPRRX1 induces ADR resistance involves the targeting of miR-3064-5p and the regulation of PTPN14. Moreover, *in vivo* experiments showed that the depletion of circPRRX1 reduced ADR resistance. In patients with GC, high levels of circPRRX1 in serum exosomes are associated with adverse effects to adriamycin treatment (88). Other studies have also found that circPVT1 and circ_0000260 are elevated in DDP-resistant GC cells and serum exosomes, and both of these circRNAs achieve DDP resistance through the ceRNA mechanism. Circ-PVT1 and circ_0000260 promote DDP resistance through the miR-30a-5p/YAP1 and miR-129-5p/MMP11 axes, respectively (89, 145). In addition, Zhong et al. found that circ_0032821 is highly expressed in oxaliplatin (OXA)-resistant GC cells and their secreted exosomes. The exosomal circ_0032821 was transmitted to OXA-sensitive cells, acting as a miR-515-5p sponge, and regulated SOX9 expression to partially enhance OXA resistance in GC cells (90).

Therefore, these exosomal ncRNAs, which can independently or supplementally regulate chemotherapy resistance in GC treatment, are promising therapeutic targets, thus having potential clinical applications.

## Potential Clinical Application of Exosomal ncRNAs in GC

### Exosomal ncRNAs Serve as Promising Biomarkers in GC

GC is one of the most lethal malignant tumors of the digestive system. The disease stage within the timeframe of diagnosis remains the predominant influential factor affecting the 5-year survival rate of patients (146). Thus, early diagnosis and treatment are crucial for improving poor prognosis. Currently, endoscopy followed by pathological examination for GC is the most reliable diagnostic tool; however, it is invasive and uncomfortable. Thus, the development of minimally invasive liquid biopsy for the diagnosis and prognosis of GC will be of great clinical significance. Liquid biopsy for marker screening requires the identification of reliable molecules for research. The stable nature of exosomes and their presence in almost all types of human biological fluids makes exosomes favorable candidates for cancer diagnosis and prognosis (147). In addition, exosomes contain various functional molecules, which can potentially serve as biomarkers or therapeutic targets (51). An increasing number of studies have found that the abnormal expression of exosomal non-coding RNAs, such as miRNAs, lncRNAs, and circRNAs, is involved in the regulation of various mechanisms of tumorigenesis and development; thus, exosomal ncRNAs have promising potential as diagnostic and prognostic markers for GC (32).

#### miRNAs

##### Diagnostic Markers

Early diagnosis and treatment are the keys to improve the prognosis of GC. The diagnostic biomarkers contribute to cancer screening and detection of tumor heterogeneity (148). Tang et al. found that higher levels of serum exosomal miR-92b-3p, let-7g-5p, miR-146b-5p, and miR-9-5p are significantly associated with early GC (stages I and II). The ability of the combined panels of exosomal miRNA or the combination of exosomal miRNA and CEA to diagnose early GC is better than that of a single exosomal miRNA marker. The exosomal miR-92b-3p + let-7g-5p + miR-146b-5p + miR-9-5p combined with CEA had the highest diagnostic efficiency, with an area under the ROC curve (AUC) value as high as 0.786 (149). Another study reported that expression levels of miR-10b-5p, miR-195-5p, miR-20a-3p, and miR-296-5p in the exosomes of GC serum samples were significantly increased (150). Shi et al. comprehensively analyzed two GEO datasets, performed qRT-PCR analysis, and found that serum miR-1246 had the largest multiple changes. Serum exosomal miR-1246 expression could distinguish TNM stage I GC patients from the healthy controls and patients with benign diseases, with AUCs of 0.843 and 0.811, respectively (151). These exosomal miRNAs can be used as potential diagnostic markers for GC, and some can be used to diagnose early GC (**Table 2**).

**Table 2:** The potential biomarker of exosomal miRNA in GC.

miRNA	Number of GC patients	Sample type	Exosome isolation techniques	Expression	Type of biomarker	Reference
miR-92b-3p, let-7g-5p, miR-146b-5p, miR-9-5p	86	Serum	ExoQuick	upregulated	Diagnostic	(149)
miR10b-5p, miR195-5p, miR20a-3p, miR296-5p	30	Serum	ExoQuick	upregulated	Diagnostic	(150)
miR-1246	85	Serum	RiboTM Exosome Isolation Reagent Kit	upregulated	Diagnostic	(151)
miR-23b	232	Plasma	ultracentrifugation	downregulated	Prognostic	(152)
miR-451	76	Serum	Total exosome isolation kit (Thermo Fisher Scientific).	upregulated	Prognostic	(130)
miR-221	40	Plasma	ExoQuick	upregulated	Prognostic	(64)
miR-3613-5p	23	Plasma	exoEasy Maxi Kit	upregulated	Prognostic	(153)
miR-379-5p, miR-410-3p	89	Plasma	ExoQuick Exosome Isolation Kit (SBI)	upregulated	Prognostic	(154)
miR-196a-1	86	Plasma	exosome isolation kit (Thermo Fisher Scientific)	upregulated	Prognostic	(70)
miR-10b-5p, miR-101-3p, miR-143-5p	126	Plasma	exoEasy Maxi Kit	upregulated	Prognostic	(155)
miR- 92a	129	Serum	ultracentrifugation	downregulated	Prognostic	(156)
miR-21	129	Serum	ultracentrifugation	upregulated	Prognostic	(156)
miR-21, miR-1225-5p	24	Peritoneal lavage fluid	ultracentrifugation	upregulated	Prognostic	(157)
miR-423-5p	80	Serum	ExoQuick	upregulated	Diagnostic and Prognostic	(158)
miR-19b-3p, miR-106a-5p	130	Serum	ExoQuick	upregulated	Diagnostic and Prognostic	(159)
miR-15b-3p	108	Serum	exoEasy Maxi Kit (Qiagen)	upregulated	Diagnostic and Prognostic	(160)

##### Prognostic Biomarkers

Prognostic biomarkers can be used to assess the risks of progression and recurrence (148). At present, the efficacy of GC treatment remains unsatisfactory, and the currently available prognostic markers for GC cannot meet the clinical needs. Thus, more accurate and effective prognostic markers of GC are required to assist in managing the treatment plan and maximizing therapeutic benefits in GC patients. Many exosomal miRNAs have been identified as promising prognostic markers.

For instance, Kumata et al. found that the level of exosomal miR-23b in GC patients is significantly lower than that in healthy controls and that the expression of miR-23b in primary tumor tissues is significantly correlated with the level of plasma exosomal miR-23b. In terms of pathological conditions, miR-23b was significantly correlated with tumor size, depth of invasion, liver metastasis, and TNM stage. Among patients with different GC staging (I–III), the overall survival (OS) rate and disease-free survival (DFS) rate of those with low exosomal miR-23 expression were significantly worse than those with high miR-23b expression. Cox multivariate analysis showed that exosomal miR-23b is an independent prognostic factor for OS and DFS at each tumor stage. Therefore, the exosomal miR-23b possesses the potential to predict the prognosis of GC patients at different stages (152). Another study showed that the serum exosomal miR-451 is significantly higher in GC patients at III–IV TNM stages and with larger tumors than in those at I–II TNM stage and with smaller tumors; in addition, the prognosis of patients in the exosomal miR 451-elevated group was significantly worse, with a 5-year survival rate of only 15.9%. Exosomal miR 451 is a more feasible and readily available indicator than tissue miR451 for predicting the prognosis of postoperative GC patients (130). Ma et al. found that the expression of miR-221 in peripheral blood exosomes is significantly upregulated in GC and that its high-expression is positively related to the poor clinical prognosis of GC, especially in tumors, lymph nodes, and metastasis stages (64).

Apart from the low diagnostic rate, high metastasis and recurrence rates also lead to a poor prognosis of GC. Many exosomal miRNAs are associated with GC metastasis and can serve as biomarkers. Chen et al. observed that the expression level of hsa-miR-3613-5p in distant metastasis samples was higher than that in healthy controls (HC) and GC without distant metastasis, with miR-3613-5p having an AUC value of 0.858 (153). Liu et al. found that the plasma exosomal miR-379-5p and miR-410-3p expressions are significantly upregulated in GC patients at stages II/III who had hematogenous metastasis after surgery. In addition, the higher expression of exosomal miR-379-5p or miR-410-3p indicated that patients had shorter progression-free survival (154).

MiR-196a-1 (70), miR-10b-5p, miR-101-3p, miR-143-5p (155) and miR-301a-3p (63) also upregulated exosomal miRNAs associated with metastasis. Feng et al. demonstrated that the plasma exosomal miR-196a-1 is higher in patients with GC than in HC. Interestingly, the stage III/IV GC patients had significantly higher expression levels of exosomal miR-196a-1 than stage I/II GC patients, indicating that exosomal miR-196a-1 is related to patients with advanced GC. Further subgroup analysis showed that the expression level of exosomal miR-196a-1 in patients with liver metastasis was significantly higher than that in patients without liver metastasis. Furthermore, the median exosomal miR-196a-1 level was used as a cut-off value to divide patients into high-level and low-level groups. The OS rate of the low-expression group was better than that of the high-expression group (70). Zhang et al. revealed that miR-10b-5p, miR-101-3p, and miR-143-5p could be used as biomarkers for GC patients with lymph node metastasis, ovarian metastasis, and liver metastasis, respectively (AUC values of 0.8919, 0.8905, and 0.8247) (155). In addition, Xia et al. found that the serum exosomal miR-301a-3p is positively correlated with peritoneal metastasis (63).

Many exosomal miRNAs are also associated with GC peritoneal recurrence. Soeda et al. observed that compared with the healthy group, the level of serum exosomal miR-21 in GC patients significantly increased, whereas that of miR-92a significantly decreased. The expression of exosomal miR-21 in patients with peritoneal recurrence was significantly higher than that in patients without peritoneal recurrence. However, the expression of exosomal miR-92a had the opposite trend. In addition, Cox multivariate analysis showed that exosomal miR-21 and miR-92a are independent prognostic factors for stage II and III GC OS and peritoneal recurrence-free survival. Therefore, they can be effective biomarkers for predicting peritoneal recurrence and prognosis in patients with stage II/III GC (156). Tokuhisa et al. investigated the differential expression of exosomal miRNAs in the peritoneal lavage fluid (PLF) of serosa invasion and non-invasive GC and verified that the expression of miR-21 and miR-1225-5p is related to the serosa infiltration of GC. Thus, exosomal miR-21 and miR-1225-5p in PLF may be used as biomarkers for peritoneal recurrence after radical resection of GC (157). In summary, these numerous exosomal miRNAs have promising prognostic potential, although only a portion of these is specific to metastasis and recurrence.

##### Diagnostic and Prognostic Markers

MiR-423-5p has been previously reported as abnormally expressed in a variety of cancers (161–163) and can promote the proliferation and invasion of GC cells (164). Yang et al. found that its high levels are associated with poor outcomes in patients with GC. Further ROC analysis revealed that exosomal miR-423-5p has higher diagnostic efficacy than traditional tumor biomarkers, such as CEA and CA-199. Its AUC was 0.763; sensitivity, 81.0%; specificity, 57.5%. On the other hand, the AUC values of serum CEA and CA-199 were 0.596 and 0.607, respectively. In addition, the level of exosomal miR-423-5p was significantly related to lymph node metastasis, and its high levels were correlated with poor prognosis in patients with GC (158). Wang et al. found that miR-19b-3p and miR-106a-5p are significantly overexpressed in patients with GC, having AUC values of 0.786 and 0.769, respectively. Their combined group also showed that the highest AUC value of 0.814 could distinguish GC patients from HC. Furthermore, these two miRNAs were associated with GC lymphatic metastasis, and their expression levels in stage III and IV patients are higher than those in stage I and II patients. Hence, miR-19b-3p and miR-106a-5p in serum exosomes are potential biomarkers for detecting GC (159).

Another study confirmed that miR-15b-3p is highly expressed in GC cell lines, tissues, and serum. Exosomes isolated from 108 GC patient serum samples and GC cell-culture medium revealed the high-expression of miR-15b-3p, with an AUC of 0.820, which is superior to those of tissues and serum miR-15b-3p (0.674 and 0.642, respectively). In addition, Kaplan–Meier analysis results showed that its high-expression in the serum could accurately predict worse OS, indicating that serum exosomal miR-15b-3p has potential as a biomarker for GC diagnosis and prognosis (160). Therefore, the aforementioned exosomal miRNAs possess the potential to become biomarkers for the diagnosis and prognosis of GC.

#### LncRNAs

##### Diagnostic Markers

GC-related long non-coding RNA1 (lncRNA-GC1) plays an important role in the occurrence of GC (165). Guo et al. detected its overexpression in GC tissues, cells, and circulating exosomes. Its diagnostic performance outperformed that of the standard biomarkers CEA, CA72-4, and CA19-9 and successfully distinguished between patients with GC and healthy donor individuals. Compared with traditional GC biomarkers (CEA, CA72-4, and CA19-9), lncRNA-GC1 exhibited the highest AUC in the early detection of GC and showed sufficient specificity and sensitivity, especially in patients with GC with negative standard biomarkers. Furthermore, the levels of circulating exosomal lncRNA-GC1 were significantly correlated with GC from early to advanced stages independent of pathological grading and Lauren classification. Therefore, exosomal lncRNA-GC1 can be used as a non-invasive biomarker to detect early GC and monitor GC progression (166).

Lin et al. identified two early gastric cancer (EGC) specific exosomal lncRNAs (lncUEGC1 and lncUEGC2) by sequencing the exosomal RNA of EGC patient plasma and GC cell-culture media. They further verified that these two lncRNAs are significantly upregulated in plasma exosomes isolated from the plasma of EGC patients and GC cell-culture media. However, stability tests have indicated that only almost all plasma lncUEGC1 is encapsulated in exosomes, thereby protecting them from RNase degradation. Furthermore, based on its AUC value, lncUEGC1 could distinguish EGC patients from HC and chronic atrophic gastritis patients (0.8760 and 0.8406, respectively). Therefore, the exosomal lncUEGC1 possesses tremendous potential as a highly sensitive, stable, and non-invasive biomarker for the diagnosis of EGC (167).

Compared with those in HC, CEBPA-AS1 and LINC00152 levels remarkably rose in the plasma exosomes of GC patients (168, 169). The AUC of plasma exosomal CEBPA-AS1 to distinguish GC from HC was calculated as 0.824, which was higher than the diagnostic accuracies of other traditional tumor biomarkers, including CEA, CA19-9, CA72-4, CA12-5, and AFP (168). In other studies, some exosomal lncRNAs, such as lnc-GNAQ-6:1, were downregulated in GC. The AUC of exosomal lnc-GNAQ-6:1 was 0.732, which was higher than the diagnostic accuracies of CEA, CA 19-9, and CA72-4 (170). Therefore, these exosomal lncRNAs are novel biomarkers with promising applications for the clinical diagnosis of GC (**Table 3**).

**Table 3:** The potential biomarker of exosomal lncRNA in GC.

LncRNA	Number of GC patients	Sample type	Exosome isolation techniques	Expression	Type of biomarker	reference
lncRNA-GC1	522	Serum	Ultracentrifugation	Upregulated	Diagnostic	(166)
lncUEGC1	51	Plasma	Ultracentrifugation	Upregulated	Diagnostic	(167)
CEBPA-AS1	281	Plasma	Ultracentrifugation	Upregulated	Diagnostic	(168)
lnc-GNAQ-6:1	43	Serum	Exosome extraction kit	Downregulated	Diagnostic	(170)
HOTTIP	246	Serum	N/A	Upregulated	Diagnostic and Prognostic	(171)
lncRNA MIAT	109	Serum	ExoQuick	Upregulated	Diagnostic and Prognostic	(172)
lncRNA H19	81	Serum	ExoQuick	Upregulated	Diagnostic and Prognostic	(173)
lnc-SLC2A12-10:1	60	Plasma	ExoQuick	Upregulated	Diagnostic and Prognostic	(174)
lncRNA ZFAS1	40	Serum	ExoQuick	Upregulated	Diagnostic and Prognostic	(175)
lncRNA SPRY4-IT1	38	Serum	Ultracentrifugation	Upregulated	Diagnostic and Prognostic	(176)

##### Diagnostic and Prognostic Markers

HOTTIP plays an important role in the occurrence and development of human cancers, such as hepatocellular carcinoma, GC, and colorectal cancer (177). Zhao et al. proposed that exosomal HOTTIP is a potential diagnostic and prognostic biomarker for GC (171). Serum exosomal HOTTIP was detected by RT-qPCR in 246 subjects (126 GC patients and 120 healthy subjects). Its expression level was generally upregulated in GC compared to HC and was significantly related to infiltration depth and TNM stage. Furthermore, exosomal HOTTIP showed higher diagnostic capability (AUC=0.827) than traditional biomarkers, including CEA, CA 19-9, and CA 72-4 (0.653, 0.685, and 0.639, respectively). Kaplan–Meier analysis demonstrated the correlation between elevated exosomal HOTTIP levels and poor OS. Moreover, univariate and multivariate Cox analyses revealed that exosomal HOTTIP overexpression is an independent prognostic factor in patients with GC.

Another study reported that the expression level of serum exosomal lncRNA MIAT in the serum of patients with GC is significantly higher than that of patients with gastric adenoma and HC. Moreover, it successfully distinguished GC patients from HC (AUC = 0.892) and gastric adenoma patients (AUC = 0.787). Interestingly, gastric adenoma patients with high serum exosomal MIAT levels are more likely to develop GC. Moreover, compared with previous surgical treatment, serum exosomal MIAT levels in blood samples have decreased significantly after treatment and increased significantly in relapsed cases. Moreover, its elevation was significantly correlated with poorer clinical variables and shorter survival time (172).

Compared with HC, lncRNA H19 and lnc-SLC2A12-10:1 in the serum exosomes of GC patients were also upregulated, and their expression levels were significantly decreased after surgery. Their AUCs were 0.849 and 0.776, respectively, which were higher than the diagnostic efficiencies of traditional diagnostic markers (CEA, CA19-9, and CA72-4). Furthermore, their expression levels were significantly correlated with the TNM stage (173, 174). Lnc-SLC2A12-10:1 was also evidently related to GC tumor size, lymph node metastasis, and degree of differentiation (174).

Other exosomal lncRNAs identified in the serum of GC patients include ZFAS1 and lncRNA SPRY4-IT1 (175, 176). Pan et al. showed that the expression level of exosomal ZFAS1 is related to lymph node metastasis and TNM stage. Its AUC, sensitivity, and specificity were calculated as 0.837, 80.0%, and 75.7%, respectively (175). Cao et al. found that the serum exosomal lncRNA SPRY4-IT1 is not only elevated in GC patients but also associated with cancer metastasis (176). These studies suggest the potential diagnostic and prognostic roles of the abovementioned exosomal lncRNAs.

#### CircRNAs

##### Diagnostic Markers

Shao et al. observed that the expression of hsa_circ_0065149 is significantly downregulated in GC but was unchanged in normal gastric mucosa, gastritis, and intestinal metaplasia. More importantly, plasma exosomes of hsa_circ_0065149 were significantly reduced in patients with EGC. Thus, they evaluated the diagnostic value of plasma hsa_circ_0065149 for EGC using a ROC curve and found that its AUC was as high as 0.640, suggesting its potential as a marker for EGC screening (178). Furthermore, compared with HC, circSHKBP1 (74) and circ-RanGAP1 (76) were significantly increased in GC tissues and peripheral blood exosomes. The levels of exosomal circSHKBP1 and circ-RanGAP1 were also evidently decreased after surgery. Notably, the plasma exosomes of GC patients containing circ-RanGAP1 promoted the migration and invasion of GC cells. In addition, exosomal circSHKBP1 promoted the growth of GC cells. Therefore, the aforementioned exosomal circRNAs have the potential to become a biomarker for the diagnosis of GC (**Table 4**).

**Table 4:** The potential biomarker of exosomal circRNA in GC.

CircRNA	Number of GC patients	Sample type	Exosome isolation techniques	Expression	Type of biomarker	reference
circ_0065149	39	plasma	Total exosome isolation reagent (Invitrogen)	Downregulated	Diagnostic	(178)
circ-RanGAP1	30	plasma	ultracentrifugation	Upregulated	Diagnostic	(76)
circSHKBP1	20	serum	ExoQuick	Upregulated	Diagnostic	(74)
circ_0000419	44	plasma	Total exosome isolation reagent (Invitrogen)	Downregulated	Diagnostic and prognostic	(179)

##### Diagnostic and Prognostic Markers

Hsa_circ_0000419 (circ_0000419) was significantly reduced in GC cell lines, tissues, and plasma of GC patients. Plasma circ_0000419 was present in exosomes and maintained good stability. Thus, its diagnostic value was investigated using ROC curves. The AUCs of tissue and plasma circ_0000419 were 0.642 and 0.840, respectively, indicating that plasma circ_0000419 is superior to tissue circ_0000419 as a biomarker for GC screening. The level of plasma circ_0000419 was significantly correlated with tumor stage, lymphatic and distal metastasis, and venous and perineural invasion (179). Thus, this study shows that circ_0000419 is a new GC screening biomarker and an important indicator for evaluating the prognosis of patients with advanced GC. In summary, these studies show that the exosomal ncRNAs in biological fluids have great potential in the development of biomarkers, and their clinical application is worthy of further study.

### The Potential Application of Exosomal ncRNA in the Treatment of GC

As bilayer phospholipid membrane capsules rich in bioactive molecules (such as ncRNAs), exosomes possess unique advantages as carriers for gene therapy. Exosomal ncRNAs play an important biological function in GC, and the strategy of specifically targeting exosomal ncRNAs may be a promising treatment option for GC patients.

Exosomes act as natural ncRNA carriers and may be suitable for delivering tumor-suppressing ncRNAs. Generally, tumor-suppressor ncRNAs are downregulated in cancer, and overexpressed ncRNAs may be encapsulated in exosomes and delivered to target cells to inhibit tumor growth. Shi et al. showed that exosomes produced by GC fibroblasts of overexpressing miRNA-34 (an anti-GC miRNA) could be absorbed by GC cells to inhibit GC cell proliferation and invasion *in vitro* and tumor growth *in vivo* (180). Xu et al. found that exosomal miR-139 transferred from CAFs to GC cells by reducing the expression of MMP11 inhibited the growth and migration of GC cells *in vitro* and suppressed metastasis of GC *in vivo* (181). Exosomal ncRNAs produced by macrophages can also inhibit GC growth. Li and colleagues found that miR-16-5p derived from M1 macrophages can be transferred to GC cells by exosomes to target PD-L1 and activate T cell immune responses, finally inhibiting GC progression (182).

For oncogenic ncRNAs, exosomes carrying inhibitors targeting such ncRNAs may reduce their expression, thereby inhibiting tumor progression. MiR-214 is dysregulated in a variety of malignant tumors, such as GC, pancreatic cancer, and lung cancer (65, 183, 184). Wang and colleagues found that the level of miR-214 in GES-1, SGC7901, and DDP-resistant SGC7901 gradually increased. Exosomes can transfer miR-214 inhibitors to GC cells and regulate potential targets, reducing cell viability, inhibiting migration, and promoting cell apoptosis *in vitro*. *In vivo* experiments demonstrated that the injection of exosomal miR-214 inhibitors into the mouse tail successfully reversed chemical resistance and inhibited tumor growth (185). In addition, Ji et al. found that the expression level of miR-374a-5p in the serum of GC increased and that this increase indicated a poor prognosis. *In vivo* and *in vitro* experiments suggest that miR-374a-5p overexpression promotes chemotherapy resistance in GC, whereas its knockdown inhibits it. Interestingly, exosomes transmit miR-374a-5p inhibitors to drug-resistant GC cells, increase the expression of Neurod1, and ultimately reverse drug resistance in GC cells both *in vitro* and *in vivo* (186). Similar studies by Wang et al. revealed that the upregulated expression of exosomal miR-221 in peripheral blood is positively correlated with poor clinical prognosis of GC. Remarkably, exosomes may act as nanocarriers to influence gene expression. Exosomes, produced by BM-MSCs transfected with miR-221 inhibitors, inhibited the proliferation, migration, invasion, and adhesion of GC cells to the matrix (64). These studies have shown that exosomes can be used as carriers to deliver anti-tumor ncRNAs or inhibitors that target oncogenic ncRNAs, thus demonstrating their potential use in therapy. However, before these treatment methods can be used in clinical practice, more efficacy and safety studies are needed.

## Conclusion and Prospect

GC is a common digestive system tumor with an existing unsatisfactory prognosis that often develops chemotherapy resistance. GC pathogenesis remains unclear and may involve many genes, signals, and molecular components. An increasing number of studies have revealed that exosomal ncRNAs play a monumental role in cell communication in the TME and possess as well as possessing the remarkable function of regulating the proliferation, invasion, metastasis, angiogenesis, immune regulation, and chemical resistance of GC, as well as other biological functions. However, so far, only a small number of exosomal ncRNAs have been confirmed, and the mechanism of other exosomal ncRNAs to promote the occurrence and development of GC needs to be further studied. How to selectively package, secrete, and transfer mechanisms of exosomal ncRNAs still need to be further explored.

Exosomal ncrnas are promising non-invasive biomarkers, which can be used for the diagnosis and prognosis monitoring of GC patients, owing to the stable nature of exosomes and their presence in almost all types of human biological fluids. However, there remains a variety of prevailing obstacles in the clinical application of exosomal ncRNAs in GC. First of all, although there exists a wide range of methods for the isolation of exosomes, it is still difficult to separate high-purity exosomes. Secondly, the ncRNAs in exosomes are limited, and how to make the most effective of them use alongside the quantification and standardization with these complex processes remains an urgent problem that awaits a viable solution. Finally, it is critical to screen candidate exosomal ncRNAs related to specific clinical parameters and clinically validate these candidate genes in a multi-center large sample cohort.

The therapeutic research of exosomal ncRNAs is still in its infancy stages of research development and clinical trials. Exosomes can also be engineered to deliver therapeutic substances(such as tumor-suppressing ncRNAs and siRNAs targeting oncogenic ncRNAs) to target cells; however, the selective distribution of therapeutic substances into exosomes and the molecular mechanism of cell targeting by exosomes remains unclear. In addition, there is still a lack of clear guidelines for the manufacture, storage, and management of treatment-related exosomes. Moreover, most studies on the role of exosomal ncRNAs in treatment only used cancer cell lines and animal models. Therefore, large-scale randomized clinical trials must be conducted on different types of cancers for further verification. With the gradual increase in understanding these unknown processes, we believe that exosomal ncRNA will become an increasingly valuable tool in disease diagnosis and treatment in the near future.

## Author Contributions

FH, YJ, and MR provided direction. FH, JL, and HL drafted the manuscript. FL, MW, and MZ revised the manuscript. All authors contributed to the article and approved the submitted version.

## Funding

The present review was supported by Special Project of Health Talents of Jilin Province (JLSWSRCZX2020-0043).

## Conflict of Interest

The authors declare that the research was conducted in the absence of any commercial or financial relationships that could be construed as a potential conflict of interest.

## Abbreviations

ALOX15, arachidonate lipoxygenase 15; BLID, BH3-like motif-containing protein; ceRNA, competing endogenous RNA; EZH2, enhancer of zeste homolog 2; FBXW7, F-box and WD repeat domain-containing 7; GATA3, GATA binding protein 3; GSDME, gasdermin E; HIF-1α, hypoxia-inducible factor1α; HMGA1, high-mobility group A1; HSP90, heat shock protein 90; HUR, Hu-antigen R; MAP7, microtubule-associated protein 7; MMP11, matrix metalloproteinase 11; NKD1, naked cuticle homolog 1; PDL1, programmed cell death ligand 1; PI3K/AKT, phosphatidylinositol 3-kinase/AKT serine/threonine kinase; PHD3, prolyl hydroxylase 3; PTEN, phosphatase and tensin homolog; PTPN14, nonreceptor tyrosine phosphatase 14; SFRP1, secreted frizzled related protein 1;Smad7, small mothers against decapentaplegic 7; SNAI1, snail family transcriptional repressor 1; SOX9, SRY-related high-mobility group box 9; STUB1, STIP1 homology and U‐Box containing protein 1; TP53INP1, tumor protein p53 to induce nucleoprotein 1; TSP-1, thrombospondin 1; *VEGF*, vascular endothelial growth factor; *VEGFA*, vascular endothelial growth factor A; YAP1, Yes-associated protein 1.
